# New Perspectives on Plant Adenylyl Cyclases

**DOI:** 10.3389/fmolb.2019.00136

**Published:** 2019-12-03

**Authors:** Oziniel Ruzvidzo, Chris Gehring, Aloysius Wong

**Affiliations:** ^1^Department of Botany, School of Biological Sciences, North-West University, Mmabatho, South Africa; ^2^Department of Chemistry, Biology and Biotechnology, University of Perugia, Perugia, Italy; ^3^Department of Biology, College of Science and Technology, Wenzhou-Kean University, Wenzhou, China

**Keywords:** *Arabidopsis thaliana*, leucine-rich repeat, adenylyl cyclase, cAMP, multidomain proteins, multiple AC centers

## Abstract

It is increasingly clear that plant genomes encode numerous complex multidomain proteins that harbor functional adenylyl cyclase (AC) centers. These AC containing proteins have well-documented roles in development and responses to the environment. However, it is only for a few of these proteins that we are beginning to understand the intramolecular mechanisms that govern their cellular and biological functions, as detailed characterizations are biochemically and structurally challenging given that these poorly conserved AC centers typically constitute only a small fraction (<10%) of complex plant proteins. Here, we offer fresh perspectives on their seemingly cryptic activities specifically showing evidence for the presence of multiple functional AC centers in a single protein and linking their catalytic strengths to the Mg^2+^/Mn^2+^-binding amino acids. We used a previously described computational approach to identify candidate multidomain proteins from *Arabidopsis thaliana* that contain multiple AC centers and show, using an *Arabidopsis* leucine-rich repeat containing protein (TAIR ID: At3g14460; AtLRRAC1) as example, biochemical evidence for multienzymatic activities. Importantly, all AC-containing fragments of this protein can complement the AC-deficient mutant *cya*A in *Escherichia coli*, while structural modeling coupled with molecular docking simulations supports catalytic feasibility albeit to varying degrees as determined by the frequency of suitable substrate binding poses predicted for the AC sites. This statistic correlates well with the enzymatic assays, which implied that the greatly reduced AC activities is due to the absence of the negatively charged [DE] amino acids previously assigned to cation-, in particular Mg^2+^/Mn^2+^-binding roles in ACs.

## Introduction

It has been established that cyclic nucleotide monophosphates, cyclic guanosine monophosphate, and cyclic adenosine monophosphate (cAMP), and their generating enzymes, guanylyl cyclases (GCs) and adenylyl cyclases (ACs), play critical roles in many diverse biological processes of living organisms ranging from prokaryotes (e.g., *Escherichia coli*) to the complex multicellular *Homo sapiens* (Moutinho et al., 2001; Newton and Smith, 2004; Schaap, 2005; Meier and Gehring, 2006; Lomovatskaya et al., 2008). It has also become clear that plant GCs (e.g., Meier et al., 2010; Qi et al., 2010; Kwezi et al., 2011; Mulaudzi et al., 2011; Irving et al., 2012; Turek and Gehring, 2016) have distinct and varied domain architectures and can be part of multifunctional enzymes or “moonlighting” proteins with two or more distinct functions (Jeffery, 2003; Irving et al., 2012; Muleya et al., 2014; Kwezi et al., 2018; Su et al., 2019). This is likely to be similar in plant ACs, whereby AC domains coexist and cofunction with other domains. Few ACs known for this include a class III AC protein (MpCAPE) in the liverwort, *Marchantia polymorpha*, with phosphodiesterase activity at its N-terminus end (Kasahara et al., 2016) and two *Arabidopsis* proteins, AtKUP5 and AtKUP7, with K^+^-uptake permease activity (Al-Younis et al., 2015, 2018).

**Figure 1:**
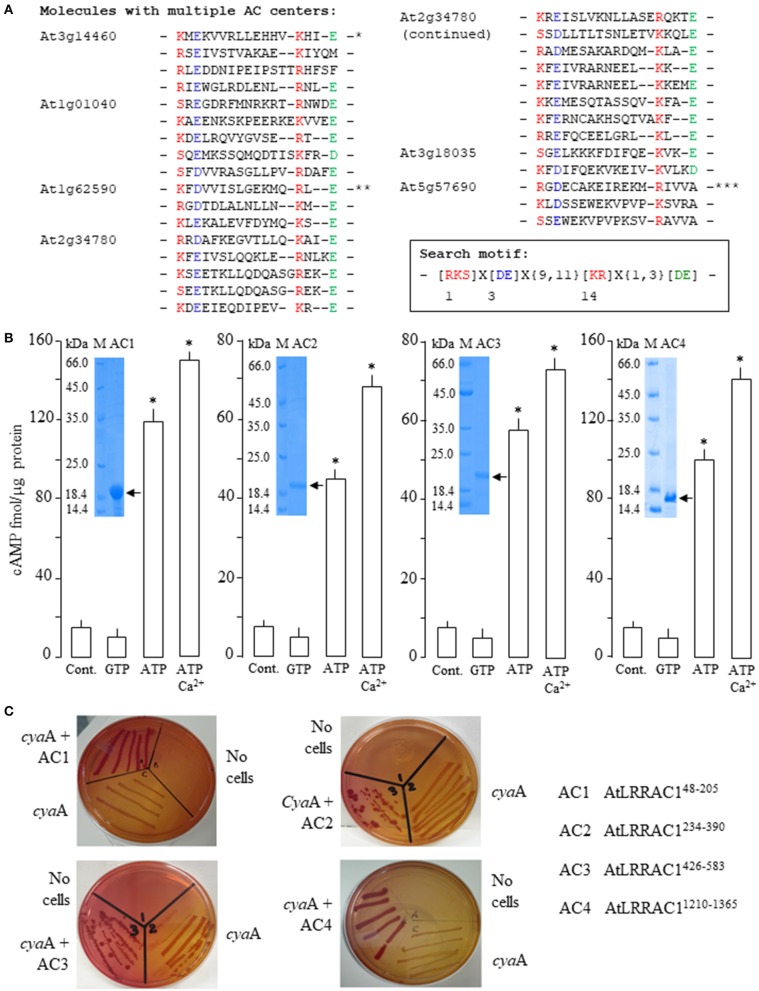
Computational identification of proteins with multiple adenylyl cyclase (AC) centers in *Arabidopsis thaliana* and functional characterizations of AtLRRAC1 AC catalytic centers. **(A)** Alignment of the AC catalytic centers of proteins with multiple AC centers in *Arabidopsis thaliana*. Asterisks denote AC centers previously confirmed to be catalytically active; *AtLRRAC1 (Ruzvidzo et al., 2013), **AtPPR-AC (Bianchet et al., 2019), and ***AtDGK4 albeit with extraordinarily high recombinant proteins used in the enzymatic assay (Dias et al., 2019). Inset: The 14-amino-acid AC search motif derived from annotated and experimentally tested guanylyl cyclases (GCs) and ACs catalytic centers. The residue forming hydrogen bonding with the purine at position 1 is highlighted in red, the residue conferring substrate specificity in position 3 is highlighted in blue, while the amino acid in position 14 that stabilizes the transition state from ATP to cyclic adenosine monophosphate (cAMP) is highlighted in red. The [DE] amino acid at one to three residues downstream from position 14 participates in Mg^2+^/Mn^2+^ binding and is colored green (Gehring, 2010). **(B)** Cyclic AMP generated by 5 μg of the AtLRRAC1^48−205^, AtLRRAC1^234−390^, AtLRRAC1^426−583^, and AtLRRAC1^1210−1365^ recombinant proteins in the presence (at final concentrations) of 1 mM ATP or GTP, or 1 mM ATP and 250 μM Ca^2+^ when 5 mM Mn^2+^ ion is the cofactor (control reaction contained all other components except the protein and Ca^2+^). Insets: Coomassie brilliant blue-stained gels after resolution of the affinity-purified His-tagged recombinant AtLRRAC1 proteins (arrows) by sodium dodecyl sulfate polyacrylamide gel electrophoresis. Data are mean values (*n* = 3), and error bars show SE of the mean. Asterisks denote values significantly different from those of the control (*P* < 0.05) as is determined by ANOVA and the *post hoc* Student–Newman–Keuls multiple range tests. **(C)** Complementation of *cya*A mutation by the AC centers of AtLRRAC1. Recombinant AtLRRAC1^48−205^, AtLRRAC1^234−390^, AtLRRAC1^426−583^, and AtLRRAC1^1210−1365^ proteins harboring the four AC centers of AtLRRAC1 complemented the *cya*A SP850 mutant *E. coli* in lactose metabolism (Shah and Peterkofsky, 1991) as indicated by the growth of deep red colonies on MacConkey agar compared to the noncomplemented *cya*A mutants that yielded yellowish colonies.

Despite the recent discoveries of plant ACs, the mechanism of actions, cellular regulations, and molecular dynamics of such catalytic centers remain largely unclear. These poorly conserved ACs retain only key residues of their catalytic centers that have been incorporated into larger multidomain proteins likely through divergent evolution (Zhang and Ma, 2012; Guo and Fang, 2014). ACs and other poorly conserved enzymes of such nature have consistently recorded low catalytic activities (Lomovatskaya et al., 2011; Hartwig et al., 2014; Gehring and Turek, 2017; Wong et al., 2018), and concerns on their biological significance were previously raised and discussed (Ashton, 2011; Berkowitz et al., 2011). Typically constituting only a small fraction of a complex plant protein (<10%), detailed characterization of AC centers becomes more challenging both biochemically and structurally (Irving et al., 2012, 2018; Wong and Gehring, 2013a; Kwezi et al., 2018). Initial efforts to identify ACs in higher plants were based on the construction of a 14-amino-acid search motif derived from annotated and experimentally tested GCs and ACs catalytic centers whereby the amino acid at position 1 forms hydrogen bonds with purine, amino acid at position 3 confers substrate specificity, and amino acid in position 14 stabilizes the transition from ATP to cAMP. The [DE] amino acids at one to three residues downstream of position 14 participates in Mg^2+^/Mn^2+^ binding (Figure 1A; Gehring, 2010). This approach has successfully identified several ACs in *Arabidopsis* many of which have since been experimentally validated, while the catalytic roles of key amino acids at the catalytic centers have also been verified by mutagenesis experiments (Gehring and Turek, 2017; Wong et al., 2018). However, the role of [DE] in regulating catalytic efficiency has never been experimentally assessed nor verified. Here, we offer fresh perspectives on the regulation of these seemingly cryptic activities specifically presenting evidence for the presence of multiple functional AC centers in a single protein and linking their catalytic strengths to the (Mg^2+^/Mn^2+^)-binding [DE] amino acids.

## AtLRRAC1 Harbors Multiple Catalytically Active AC Centers that Complemented AC-Deficient *E. coli*

Applying a previously detailed computational approach to identify candidate AC domains in proteins from *Arabidopsis thaliana* (Ludidi and Gehring, 2003; Gehring, 2010; Wong and Gehring, 2013a; Wong et al., 2018), the *At3g14460* gene was shown to encode a leucine-rich repeat (LRR) containing protein that harbors four distinct AC centers (Figure 1A), which are spatially distributed throughout the entire protein (Supplementary Figure 1). The AC centers are referred to as AC1, AC2, AC3, and AC4, respectively, and were tested individually for catalytic activity. Besides At3g14460, *A. thaliana* also harbors five other proteins with multiple AC centers: At1g01040 with 5, At1g62590 with 3, At2g34780 with 13, At3g18035 with 2, and At5g57690 with 3 centers (Figure 1A). Notably, At3g14460 (AtLRRAC1), At1g62590 (AtPPR-AC), and At5g57690 (AtDGK4) (Figure 1A) have been experimentally shown to be functional ACs (Ruzvidzo et al., 2013; Bianchet et al., 2019; Dias et al., 2019).

After the identification of the four AC centers in AtLRRAC1, each center was then separately cloned, expressed, and affinity purified to produce four respective truncated recombinant proteins: AtLRRAC1^48−205^, AtLRRAC1^234−390^, AtLRRAC1^426−583^, and AtLRRAC1^1210−1365^ (Figure 1B, insets). When these truncated protein fractions were tested *in vitro* for their ability to convert ATP to cAMP, they all demonstrated significant Mn^2+^-dependent AC activities that could be enhanced by Ca^2+^ (Figure 1B). Incidentally, Ca^2+^-dependent activity increases in plant GCs and ACs have been observed previously (Muleya et al., 2014; Wheeler et al., 2017; Chatukuta et al., 2018), and in GCs, Ca^2+^ can act as switch between the kinase and GC activities of the dual-functioning phytosulfokine receptor (AtPSKR1) (Muleya et al., 2014). However, when comparing the *in vitro* AC activities of the four AC centers of AtLRRAC1, the activities of AtLRRAC1^234−390^ and AtLRRAC1^426−583^ were approximately half those of AtLRRAC1^48−205^ and AtLRRAC1^1210−1365^ (Figure 1B). We speculate that this reduction in activity is due to the absence of the negatively charged [DE] amino acids downstream of the AC catalytic centers of AtLRRAC1^234−390^ and AtLRRAC1^426−583^ (marked green in Figure 1A) responsible for Mg^2+^/Mn^2+^ binding (Ludidi and Gehring, 2003; Gehring, 2010).

To further support our *in vitro* AC activity findings, each of the four AC centers of AtLRRAC1 was tested to see if it could rescue the AC-deficient *E. coli* mutant strain SP850 with a *cya*A mutation essential for lactose fermentation. When each of the AC centers was cloned and expressed in the SP850 mutant followed by growth on MacConkey agar, cells turned deep red, indicating a rescue to the wild-type phenotype (Figure 1C). This result corresponds well with the biochemical assay, which also showed catalytic activity in all four AC-containing recombinant proteins (Figure 1B).

## AC Centers Harboring Negatively Charged Mg^2+^/Mn^2+^-Binding [DE] Amino Acids Docked with ATP at Higher Correct Binding Pose Frequencies

**Figure 2:**
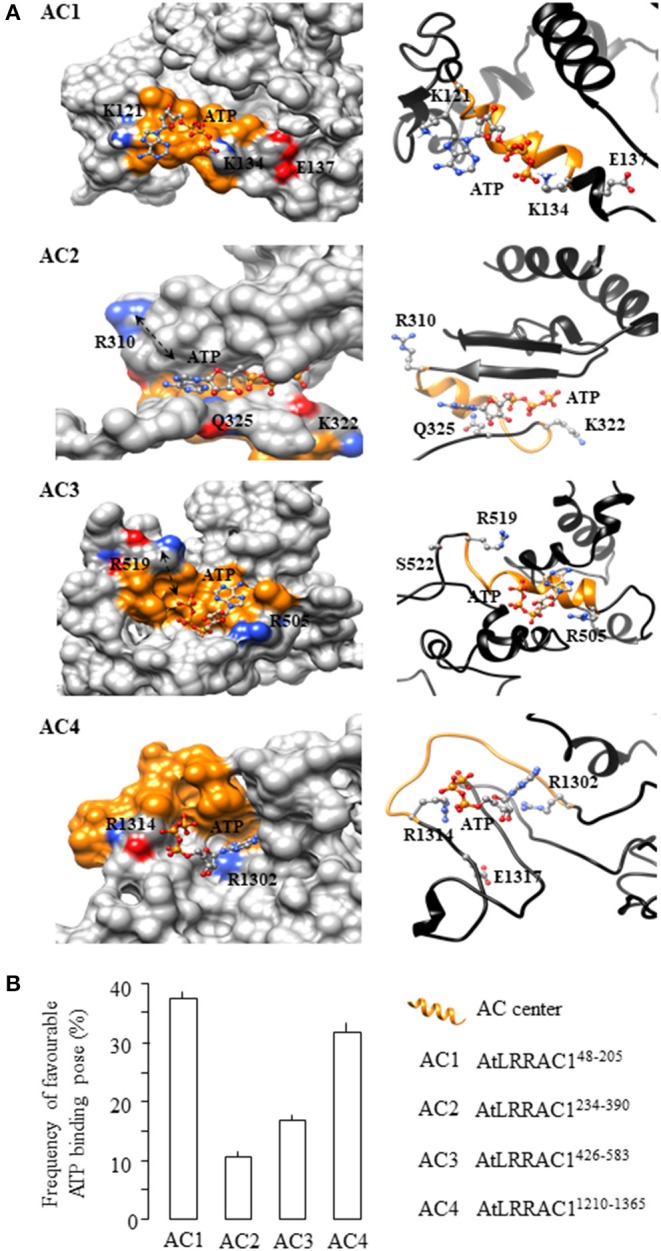
Computational assessment of the adenylyl cyclase (AC) centers of AtLRRAC1. **(A)** Representative images of the four AC-containing fragments of AtLRRAC1 (AtLRRAC1^48−205^, AtLRRAC1^234−390^, AtLRRAC1^426−583^, and AtLRRAC1^1210−1365^) docked with ATP are shown as AC1, AC2, AC3, and AC4, respectively. The interaction of ATP with key residues at the AC catalytic centers of each fragment is shown as surface (left panel) and ribbon models (right panel), respectively. The amino acid residues at positions 1 and 14 of the AC centers, which are implicated in interactions with ATP, are colored according to their charges in the surface models and shown as individual atoms in the ribbon models. AC centers are colored orange, and black arrows in the surface models of AtLRRAC1^234−390^ and AtLRRAC1^426−583^ show the far distance between ATP and the key amino acids in the AC centers than in AtLRRAC1^48−205^ and AtLRRAC1^1210−1365^. Full-length AtLRRAC1 model was generated using the iterative threading assembly refinement (I-TASSER) method on the online server: http://zhanglab.ccmb.med.umich.edu/I-TASSER/ (Zhang, 2008), and ATP docking simulations were performed using AutoDock Vina (ver. 1.1.2) (Trott and Olson, 2010). Molecular graphics and analyses were performed with the UCSF Chimera package (Pettersen et al., 2004). **(B)** Frequency of favorable ATP binding pose at the AC centers of AtLRRAC1 as estimated by molecular docking. All four AC-containing fragments of the AtLRRAC1 (AtLRRAC1^48−205^, AtLRRAC1^234−390^, AtLRRAC1^426−583^, and AtLRRAC1^1210−1365^) were docked with ATP at their AC centers, and a total of 18 solutions generated by AutoDock Vina (ver. 1.1.2) (Trott and Olson, 2010) for each fragment were then evaluated and expressed as percentage. All clusters, docking data, and interpretation of docking solutions are provided in Supplementary Figure 2. Orientations and binding poses were analyzed with the UCSF Chimera package (Pettersen et al., 2004). Chimera is developed by the Resource for Biocomputing, Visualization, and Informatics at the University of California, San Francisco (supported by NIGMS P41-GM103311).

We then examined the structures of the four AC-containing fragments of AtLRRAC1 by iterative threading assembly (Zhang, 2008) followed by molecular docking of ATP to their AC centers using AutoDock Vina (Trott and Olson, 2010) and evaluated the binding poses and their free energies. We observed that ATP docked to AtLRRAC1^48−205^ and AtLRRAC1^1210−1365^ with its adenine and phosphate ends situated spatially close to the key amino acids of the AC centers (Figure 2A). However, in AtLRRAC1^234−390^ and AtLRRAC1^426−583^, ATP docked in a manner that its adenine or phosphate appears to be too distant for interactions with one of the key amino acids at the AC centers (see black arrows in Figure 2A). In addition to this spatial consideration, we also analyzed the orientations of docked ATP for “correct binding pose” according to previously ascertained deductions and rationales (Wong and Gehring, 2013b; Wong et al., 2015). Here, we define a “correct binding pose” as ATP orientation with its adenine head pointing toward the amino acid at position 1 and its phosphate tail pointing toward the positively charged amino acid at position 14 of the AC motif (Figure 1A inset; Supplementary Figure 2)–a definition that is consistent with that of previous works on plant ACs and GCs (Wong and Gehring, 2013b; Wong et al., 2015). This orientation of nucleotides has been deemed favorable for catalysis in previously characterized ACs and GCs (Al-Younis et al., 2015, 2018; Wheeler et al., 2017; Chatukuta et al., 2018; Bianchet et al., 2019) where, notably, mutagenesis of these residues have yielded reduced catalytic activities (Wheeler et al., 2017; Al-Younis et al., 2018). To evaluate the likelihood of the “correct ATP binding pose” at the AC centers of the AtLRRAC1 protein, a total of 18 solutions generated by AutoDock Vina were evaluated and expressed as percentage. We discovered that docking of ATP to the AC1 center yielded the highest “correct binding pose” frequency of 38.9% compared to the other AC centers. The “correct binding pose” frequencies for the other AC centers were 33.3% for the AC4 center, 16.7% for the AC3 center, and 11.1% for the AC2 center (Figure 2B). In AC2 and AC3 centers, even in instances where the “correct binding pose” was obtained, part of the ATP substrate (adenine or phosphate) seems too far from the key amino acids at the AC centers as indicated by black arrows in the surface models (Figure 2A; Supplementary Figure 2). Once again, this finding is consistent with our *in vitro* biochemical results that showed that the AtLRRAC1^48−205^ and AtLRRAC1^1210−1365^ recombinants generate approximately twice as much cAMP than the AtLRRAC1^234−390^ and AtLRRAC1^426−583^ recombinants (Figure 1B). All clusters, docking data and interpretation of docking solutions are provided in Supplementary Figure 2. Modeling and docking experiments have provided visualizations of how the respective catalytic centers could accommodate ATP. Furthermore, docking statistics not only provided valuable support to our biochemical data but also increased the degree of confidence of both the experimental results and our proposed roles for the [DE] residues in the motif. However, at this point, we are unsure how the absence of [DE] resulted in poorer ATP docking poses in AC2 and AC3, but we speculate that absence of [DE] resulted in loosened substrate binding pockets.

Taken together, structural assessments of the AC centers in AtLRRAC1 showed that the AtLRRAC1^234−390^ and AtLRRAC1^426−583^ fragments accommodated ATP less favorably (both spatially and in terms of frequency of the “correct binding pose”) compared to the AtLRRAC1^48−205^ and AtLRRAC1^1210−1365^ fragments. Again, it is tempting to speculate that this might be due to the absence of the negatively charged [DE] amino acids downstream of the AC catalytic centers of the AtLRRAC1^234−390^ and AtLRRAC1^426−583^ fragments (marked green in Figure 1). These amino acids appear at two amino acids downstream of the AC centers of the AtLRRAC1^48−205^ and AtLRRAC1^1210−1365^ fragments, and they normally appear at one to three amino acids downstream of the AC centers and have been previously assigned with the role of cation binding and in particular Mg^2+^ or Mn^2+^ (e.g., Gehring, 2010). Previous AC searches and characterizations have excluded hits without [DE] (Wong and Gehring, 2013b; Gehring and Turek, 2017; Wong et al., 2018), although the more recently created AC/GC prediction tools, ACPred and GCPred, offer the option for excluding [DE] in their servers (Xu et al., 2018a,b) to provide greater flexibility. Divalent cation (Mg^2+^/Mn^2+^) binding residues [DE] are known to coordinate the phosphates of ATP/ADP or GTP/GDP to the catalytic site of various enzymes including but not limited to kinases, ATP-/GTP-ases and phosphodiesterases. Divalent cation (Mg^2+^/Mn^2+^) binds to conserved [DE] amino acids in bacterial- and animal-soluble GCs typically at one to three residues downstream of the catalytic centers. Our AC and GC motifs were initially constructed by extracting key functional amino acids which are conserved at catalytic sites of canonical ACs and GCs across species, including the [DE] amino acids. Our motifs have since identified several ACs and GCs, many of which have been experimentally validated, but the role of [DE] in regulating their catalytic efficiency has never been assessed experimentally. Here, we showed for the first time that AC centers missing the [DE] residues have significantly reduced activities, and these preliminary data could explain past results, e.g., AtDGK4 by Dias et al. (2019) and/or guide future characterization works.

## Discussion and Outlook

The possibility of proteins harboring multiple catalytically active AC centers is intriguing as it adds yet another layer of complexity to the regulation of signaling pathways in plant cells (Wong et al., 2015; Marondedze et al., 2017; Irving et al., 2018; Kwezi et al., 2018; Swiezawska et al., 2018; Su et al., 2019). Multiple functional AC centers in a single protein may also offer an additive effect which can accumulatively increase the catalytic strength. If these catalytic centers are proven to be functional *in vivo*, questions such as “are the centers catalytically active at the same time?” “how are their activities regulated?” and “do they cross-talk?” will require further investigations *in vivo* and *in planta*. Interestingly, a recent work by Dias et al. (2019) showed that the *Arabidopsis thaliana* DGK4 protein has GC but not AC activity *in vitro* with 10 or 40 μg recombinant protein. AC activity was only detected when the recombinant protein was raised to an unphysiologically high amount of 400 μg. Notably, this protein has a nucleotide cyclase center with the [DE] Mg^2+^/Mn^2+^-binding residue, and although it has three AC centers (Figure 1A), they all lacked the [DE] amino acids which might account for the weakened AC activity. From physiological studies, the authors have assigned this protein to various signaling roles regulating lipid composition, cytoskeletal dynamics, and pollen tube growth that affected fertilization *in planta*. Since the AC centers in DGK4 satisfied our AC motif (Figure 1A) and was also predicted by ACPred (Xu et al., 2018b), these could be indirect evidence for the molecular and biological roles of [DE] in GC/AC centers of such nature. Further mutagenesis experiments that add or remove the [DE] amino acids from the respective AC centers of AtLRRAC1 will validate our current data. We also hypothesize that the multidomain ACs might be capable of forming intramolecular dimers that in turn affect catalytic activities and, consequently, cAMP-dependent downstream signaling. In conclusion, our evidence for the presence of multiple functional AC centers in a single protein and linking catalytic strength to the Mg^2+^/Mn^2+^-binding [DE] amino acids has offered fresh perspectives that will contribute to the eventual goal of elucidating the intricate molecular regulations of such poorly conserved catalytic centers.

## Materials and Methods

### Generation of the AtLRRAC1 Recombinant Proteins

Total RNA was extracted from 6-week-old *A. thaliana* ecotype Columbia-0 (Col-0) seedlings using the RNeasy plant mini kit, in combination with DNase 1 treatment, as instructed by the manufacturer (Qiagen, Crawley, UK). Copy DNA (cDNA) sequence of *AtLRRAC1* or At3g14460 was retrieved from The *Arabidopsis* Information Resource (TAIR) (https://www.arabidopsis.org) and checked for presence of multiple AC catalytic centers using the PROSITE database located within the Expert Protein Analysis System (ExPASy) proteomics server (https://www.expasy.org). cDNA synthesis from the total RNA and subsequent amplification of each of the identified multiple AC catalytic centers from the cDNA were simultaneously performed in the presence of a set of each of the respective sequence-specific primer pairs in Supplementary Table 1, using a Verso 1-Step RT-PCR kit and in accordance with the manufacturer's instructions (Thermo Scientific, Rockford, USA). The PCR products were then cloned into a pTrcHis2-TOPO expression vector via the TA cloning system (Invitrogen Corp., Carlsbad, USA) to make pTrcHis2-TOPO:*AtLRRAC1* fusion expression constructs with C-terminus His purification tags. Expression, purification, and refolding processes of the recombinant AtLRRAC1 proteins were undertaken as is detailed elsewhere (Meier et al., 2010; Kwezi et al., 2011; Chatukuta et al., 2018) and their relative molecular masses estimated using the ProtParam tool on the ExPasy Proteomics Server (http://au.expasy.org/tool/.protpatram.html). The purified proteins were then used for *in vitro* enzymatic assays.

### *In vitro* AC Enzymatic Assays and Detection of cAMP

The AC activity of each of the purified recombinant AtLRRAC1 proteins were determined *in vitro* by incubating 5 μg of the protein in 50 mM Tris–Cl buffer (pH 8.0), containing at, final concentration, 5 mM Mn^2+^, 1 mM ATP or 1 mM GTP, and 2 mM 3-isobutyl-1-methylxanthine, followed by measurement of the generated cAMP. Since Ca^2+^ has been shown to enhance the activity of ACs (Chatukuta et al., 2018) or GCs and indeed acting as a cellular switch between the GC and kinase activities of AtPSKR1 (Muleya et al., 2014), it was also tested against the AtLRRAC1 recombinants at a final concentration of 250 μM and in the presence of 5 mM Mn^2+^, 1 mM ATP, and 2 mM 3-isobutyl-1-methylxanthine. Levels of the generated cAMP were determined by enzyme immunoassaying following its acetylation protocol as described by the supplier's manual (Sigma-Aldrich Corp., Missouri, USA; code: CA201). The methods are detailed elsewhere (Ruzvidzo et al., 2013).

### Complementation of *cya*A Mutation in the *E. coli* AC-deficient Strain

The *E. coli cya*A mutant SP850 strain [lam-, el4-, relA1, spoT1, *cya*A1400 (:kan), thi-1] (Shah and Peterkofsky, 1991), deficient in the adenylyl cyclase (*cya*A) gene, was obtained from the *E. coli* Genetic Stock Centre (Yale University, New Haven, USA) (accession no. 7200). The strain was prepared to be chemically competent followed by its transformation with the different pTrcHis2-TOPO:*AtLRRAC1* fusion constructs (through heat shock at 42°C for 2 min). The transformed bacteria were then grown at 37°C for 18–40 h on MacConkey agar supplemented with 1% lactose, 15 μg/ml kanamycin, and 0.5 mM of the transgene inducer, isopropyl-β-d-thiogalactopyranoside (Sigma-Aldrich Corp., Missouri, USA) (Moutinho et al., 2001; Ruzvidzo et al., 2013; Swiezawska et al., 2015).

### Computational Assessment of the AtLRRAC1 Multiple AC Catalytic Centers

An AtLRRAC1 model was generated using the iterative threading assembly refinement (I-TASSER) method (Zhang, 2008). The full-length AtLRRAC1 amino acid sequence (1,401 aa) was submitted to the I-TASSER server available online at http://zhanglab.ccmb.med.umich.edu/I-TASSER/, and a model with the highest *C*-score was downloaded from the server and used for the subsequent molecular docking experiments. Docking of ATP to AC centers of the various AtLRRAC1 fragments, AtLRRAC1^48−205^, AtLRRAC1^234−390^, AtLRRAC1^426−583^, and AtLRRAC1^1210−1365^ was performed using AutoDock Vina (ver. 1.1.2) (Trott and Olson, 2010). The AC centers of the various AtLRRAC1 fragments and ATP docking poses were analyzed and all images created by UCSF Chimera (ver. 1.10.1) (Pettersen et al., 2004). Chimera is developed by the Resource for Biocomputing, Visualization, and Informatics at the University of California, San Francisco (supported by NIGMS P41-GM103311). To evaluate the frequency of the “correct ATP binding pose” at the AC centers, a total of 18 solutions generated by AutoDock Vina were evaluated and expressed as percentage.

### Statistical Analysis

All data of immunoassay in this work was subjected in triplicates (*n* = 3) to analysis of variance (ANOVA) (Super-Anova, Statgraphics Version 7, Statgraphics Corporation, USA). Where ANOVA revealed significant differences between treatments, means were separated by *post hoc* Student–Newman–Keuls multiple range test (*P* < 0.05).

## Data Availability Statement

The datasets generated for this study are available on request to the corresponding author.

## Author Contributions

OR, CG, and AW conceived and designed the study. OR performed the functional characterizations. AW undertook the structural works. All authors wrote the manuscript and read and approved its final version.

### Conflict of Interest

The authors declare that the research was conducted in the absence of any commercial or financial relationships that could be construed as a potential conflict of interest.
