# Supplementary material for: New Perspectives on Plant Adenylyl Cyclases
Source: Front Mol Biosci. 2019 Dec 3;6:136. doi: 10.3389/fmolb.2019.00136 (PMC6901789; doi:10.3389/fmolb.2019.00136)
Supplement: Supplementary file 1 [file Data_Sheet_1.pdf]

Supplementary Material

1 Supplementary Figure

```
1                                                                                                  100
MANSYLSSCANVMVERINTSQELVELCKGKSSSALLKRLKVALVTANPVLADADQRAEHVREVKHWLTGIKDAFFQAEDILDELQTEALRRRVVAEAGGL
GGLFQNLMAGREAIQKKIEPKMEKVVRLLEHHVKHIEVIGLKEYSETREPQWRQASRSRPDDLPQGRLVGRVEDKLALVNLLLSDDEISIGKPAVISVVG
MPGVGKTTLTEIVFNDYRVTEHFEVKMWISAGINFNVFTVTKAVLQDITSSAVNTEDLPSLQIQLKKTLSGKRFLLVLDDFWSESDSEWESFQVAFTDAE
EGSKIVLTTRSEIVSTVAKAEKIYQMKLMTNEECWELISRFAFGNISVGSINQELEGIGKRIAEQCKGLPLAARAIASHLRSKPNPDDWYAVSKNFSSYT
NSILPVLKLSYDSLPPQLKRCFALCSIFPKGHVFDREELVLLWMAIDLLYQPRSSRRLEDIGNDYLGDLVAQSFFQRLDITMTSFVMHDLMNDLAKAVSG
DFCFRLEDDNIPEIPSTTRHFSFSRSQCDASVAFRSICGAEFLRTILPFNSPTSLESLQLTEKVLNPLLNALSGLRILSLSHYQITNLPKSLKGLKLLRY
LDLSSTKIKELPEFVCTLCNLQTLLLSNCRDLTSLPKSIAELINLRLLDLVGTPLVEMPPGIKKLRSLQKLSNFVIGRLSGAGLHELKELSHLRGTLRIS
ELQNVAFASEAKDAGLKRKPFLDGLILKWTVKGSGFVPGSFNALACDQKEVLRMLEPHPHLKTFCIESYQGGAFPKWLGDSSFFGITSVTLSSCNLCISL
PPVGQLPSLKYLSIEKFNILQKVGLDFFFGENNSRGVPFQSLQILKFYGMPRWDEWICPELEDGIFPCLQKLIIQRCPSLRKKFPEGLPSSTEVTISDCP
LRAVSGGENSFRRSLTNIPESPASIPSMSRRELSSPTGNPKSDASTSAQPGFASSSQSNDDNEVTSTSSLSSLPKDRQTEDFDQYETQLGSLPQQFEEPA
VISARYSGYISDIPSTLSPYMSRTSLVPDPKNEGSILPGSSSYQYHQYGIKSSVPSPRSSEAIKPSQYDDDETDMEYLKVTDISHLMELPQNLQSLHIDS
CDGLTSLPENLTESYPNLHELLIIACHSLESFPGSHPPTTLKTLYIRDCKKLNFTESLQPTRSYSQLEYLFIGSSCSNLVNFPLSLFPKLRSLSIRDCES
FKTFSIHAGLGDDRIALESLEIRDCPNLETFPQGGLPTPKLSSMLLSNCKKLQALPEKLFGLTSLLSLFIIKCPEIETIPGGGFPSNLRTLCISLCDKLT
PRIEWGLRDLENLRNLEIDGGNEDIESFPEEGLLPKSVFSLRISRFENLKTLNRKGFHDTKAIETMEISGCDKLQISIDEDLPPLSCLRISSCSLLTETF
AEVETEFFKVLNIPYVEIDGEIFS
```

Supplementary Figure 1. Full amino acid sequence the AtLRRAC1 (1401 amino acids) with its 4 AC catalytic centres highlighted in bold and underlined. The four respective truncated versions of the AtLRRAC1 protein (AtLRRAC1$^{48-205}$, AtLRRAC1$^{234-390}$, AtLRRAC1$^{426-583}$ and AtLRRAC1$^{1210-1365}$) expressed and tested for AC activity in this study are marked within the inverted red triangles.

AC1 docking solutions							
Mode 1st run	Affinity	Distance from best mode		Mode 2nd run	Affinity	Distance from best mode	
	(kcal/mol)	rmsd l.b.	rmsd u.b.		(kcal/mol)	rmsd l.b.	rmsd u.b.
1	-4.1	0.000	0.000	1	-3.7	0.000	0.000
2	-4.1	5.064	7.509	2	-3.5	5.021	7.585 ✓
3	-3.9	1.614	2.196	3	-3.4	5.050	7.499 ✓
4	-3.8	1.660	2.322	4	-3.3	2.199	2.857
5	-3.6	4.965	6.778 ✓	5	-3.1	4.415	6.429 ✓
6	-3.1	3.925	6.662 ✓	6	-2.7	1.792	2.217
7	-3.1	5.484	8.118	7	-2.3	2.046	3.148
8	-2.9	2.639	4.337 ✓	8	-2.2	5.259	7.560 ✓
9	-2.6	4.777	7.161	9	-2.1	4.095	6.910

✓ = selected solutions with "correct binding pose" where adenine points towards (→) K131 and phosphate → K134

Total "correct binding pose" (✓) = 7/18 = 38.9 %

AC1 correct ATP binding pose (7 ✓)

AC1 incorrect ATP binding pose (11)

1st run

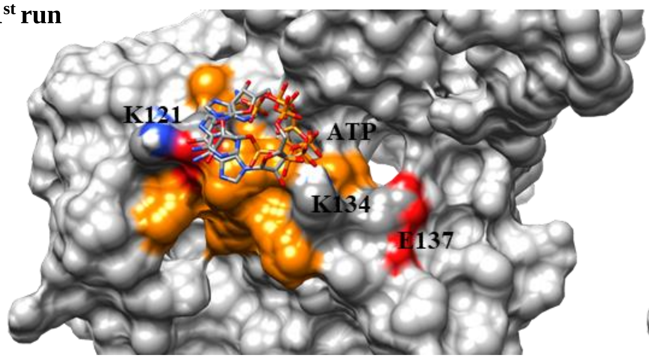

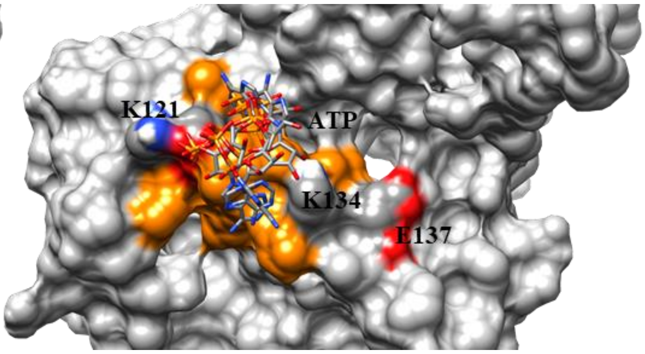

2nd run

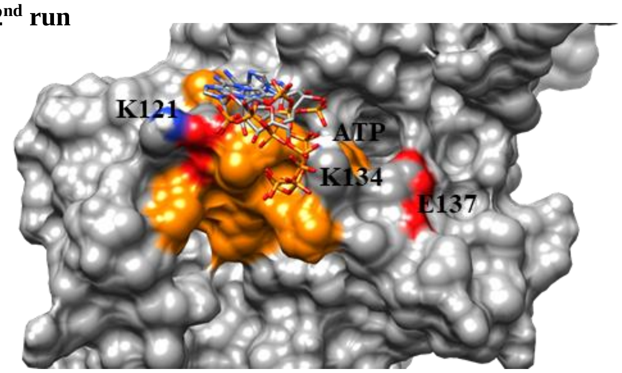

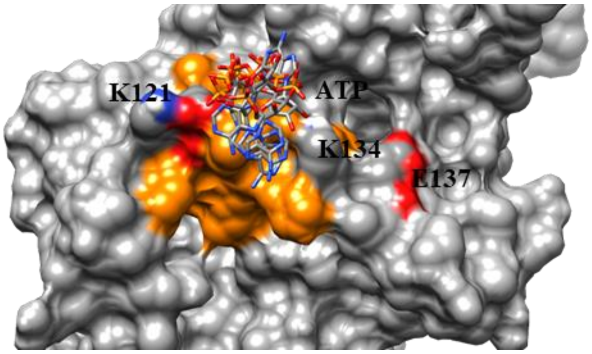

AC2 docking solutions							
Mode<br>1st run	Affinity	Distance from best mode		Mode<br>2nd run	Affinity	Distance from best mode	
	(kcal/mol)	rmsd l.b.	rmsd u.b.		(kcal/mol)	rmsd l.b.	rmsd u.b.
1	-5.7	0.000	0.000	1	-6.4	0.000	0.000
2	-5.3	0.971	1.891	2	-6.1	1.239	2.120
3	-5.3	3.562	5.808	3	-6.0	2.404	5.427 ✓
4	-5.0	1.544	2.291	4	-5.9	4.456	6.464
5	-4.8	3.842	6.111 ✓	5	-5.6	1.548	2.895
6	-4.7	1.233	2.151	6	-5.3	2.803	4.532
7	-4.6	3.874	6.144 ✓	7	-5.1	3.490	5.343
8	-4.4	1.404	2.546	8	-5.0	1.955	3.548
9	-4.3	4.250	7.064	9	-5.0	2.699	4.811

✓ = selected solutions with "correct binding pose" where adenine points towards (→) R310 and phosphate → K322

Total "correct binding pose" (✓) = 2/18 = 11.1 %

AC2 correct ATP binding pose (2 ✓)

AC2 incorrect ATP binding pose (16)

1st run

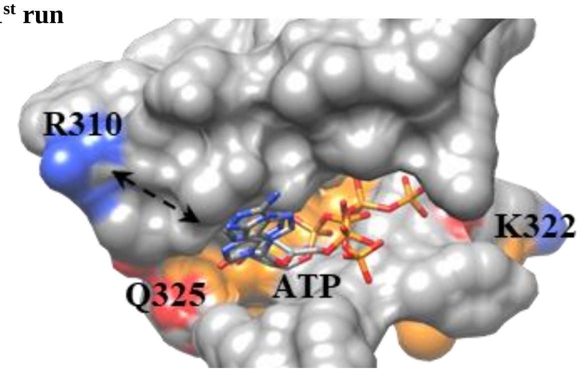

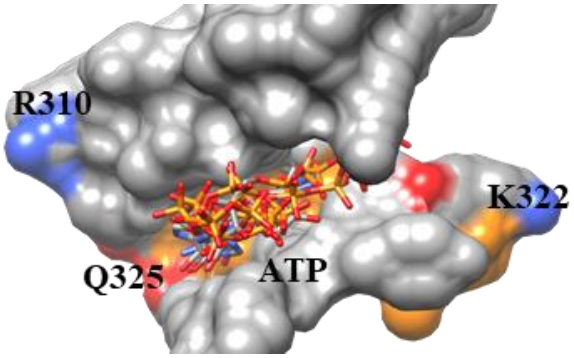

2nd run

No "correct binding pose" found

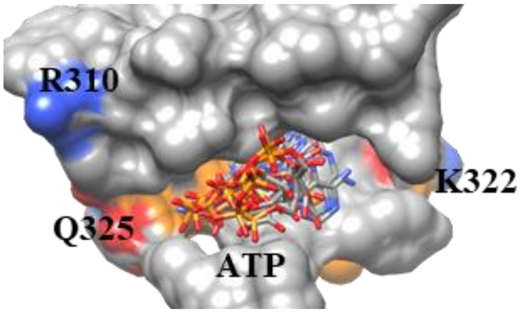

AC3 docking solutions							
Mode 1st run	Affinity	Distance from best mode		Mode 2nd run	Affinity	Distance from best mode	
	(kcal/mol)	rmsd l.b.	rmsd u.b.		(kcal/mol)	rmsd l.b.	rmsd u.b.
1	-6.2	0.000	0.000	1	-6.6	0.000	0.000
2	-6.1	2.916	5.321	2	-6.6	2.660	4.909 ✓
3	-6.0	2.339	4.020 ✓	3	-6.5	2.585	5.318
4	-5.9	1.745	2.072	4	-6.5	1.404	2.231
5	-5.7	1.977	3.626	5	-6.5	1.571	2.235
6	-5.6	3.634	6.258	6	-6.4	1.738	3.538
7	-5.6	1.978	3.987	7	-6.3	2.378	4.868
8	-5.6	1.665	3.089 ✓	8	-6.2	2.560	3.819
9	-5.5	3.404	6.196	9	-6.2	2.329	4.927

✓ = selected solutions with “correct binding pose” where adenine points towards (→) R505 and phosphate → R519

Total **“correct binding pose” (✓)** = 3/18 = 16.7 %

AC3 correct ATP binding pose (3 ✓)

AC3 incorrect ATP binding pose (15)

1st run

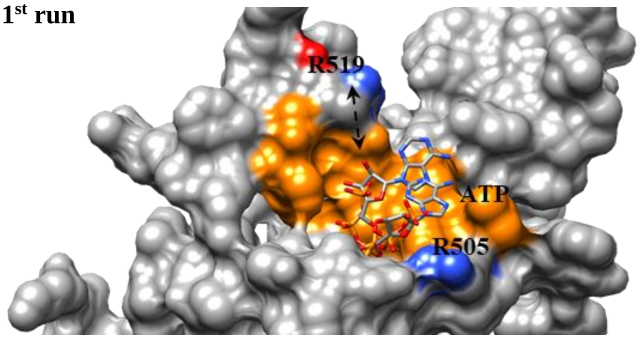

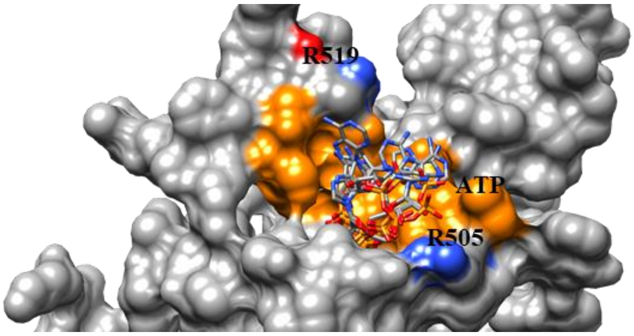

2nd run

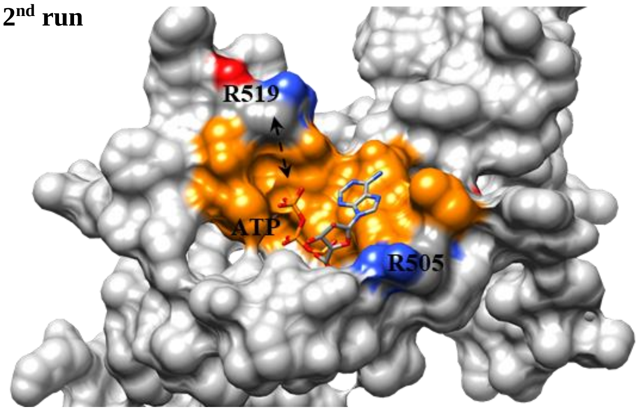

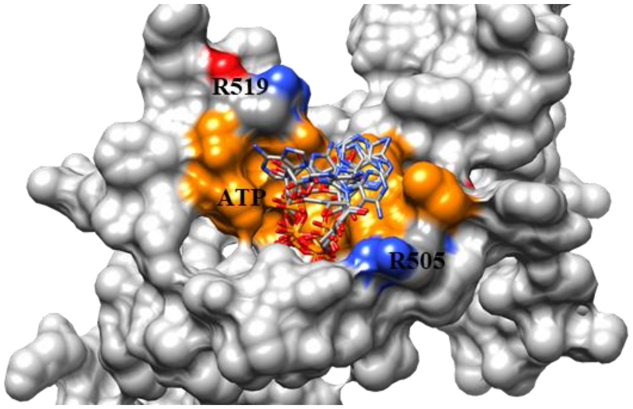

AC4 docking solutions							
Mode	Affinity	Distance from best mode		Mode	Affinity	Distance from best mode	
1st run	(kcal/mol)	rmsd l.b.	rmsd u.b.	2nd run	(kcal/mol)	rmsd l.b.	rmsd u.b.
1	-6.5	0.000	0.000	1	-6.4	0.000	0.000
2	-6.2	6.086	8.862 ✓	2	-6.0	2.066	3.731
3	-6.1	1.780	2.836	3	-6.0	3.836	6.377
4	-6.0	1.727	3.050	4	-5.8	1.627	2.925
5	-5.7	3.943	6.506	5	-5.8	1.609	2.790
6	-5.7	3.177	6.016	6	-5.8	5.686	9.028 ✓
7	-5.7	1.495	2.260	7	-5.7	5.780	8.221 ✓
8	-5.6	6.135	9.976 ✓	8	-5.5	5.311	8.585 ✓
9	-5.6	5.679	8.764 ✓	9	-5.5	1.945	3.203

✓ = selected solutions with "correct binding pose" where adenine points towards (→) R1302 and phosphate → R1314

Total "correct binding pose" (✓) = 6/18 = 33.3 %

AC4 correct ATP binding pose (6 ✓) **AC4 incorrect ATP binding pose (12)**

1st run

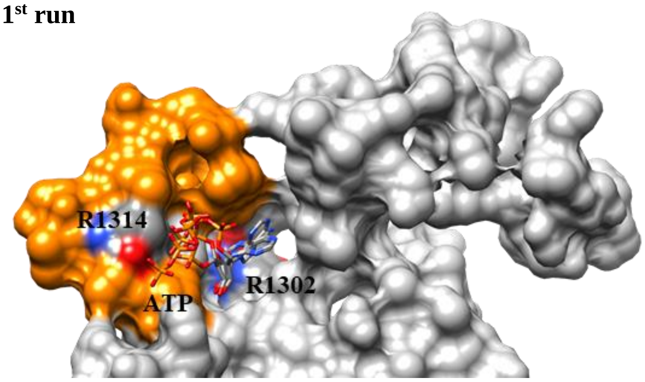

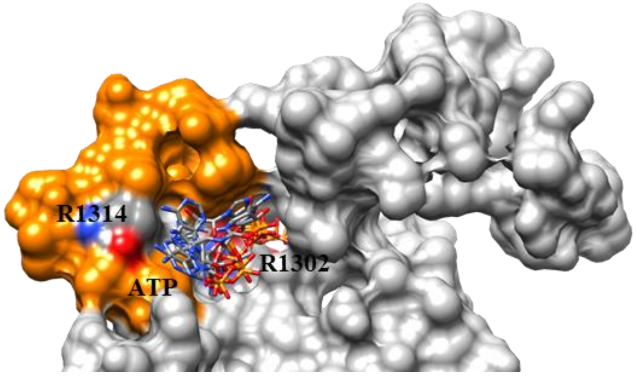

2nd run

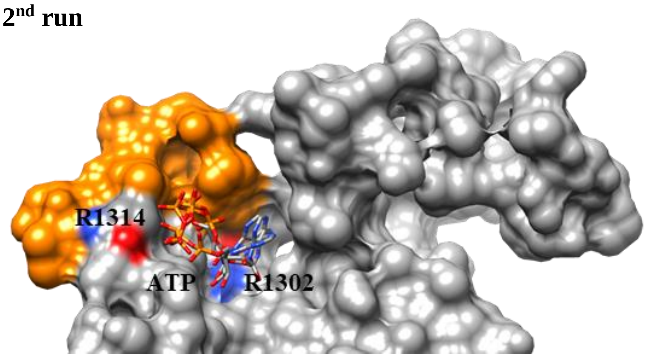

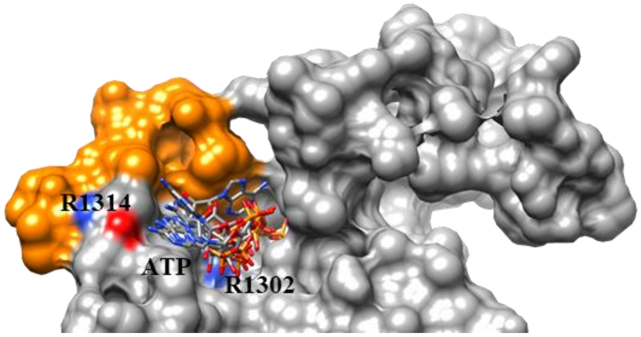

Supplementary Figure 2. Clusters, docking data of all four AC fragments in AtLRRAC1 and interpretation of the docking solutions. All AC models were generated using the iterative threading assembly refinement (I-TASSER) method on the on-line server: http://zhanglab.ccmb.med.umich.edu/I-TASSER/ (Zhang, 2008) and ATP docking simulations were performed using AutoDock Vina (ver. 1.1.2) (Trott and Olson, 2010). All four AC-containing fragments of the AtLRRAC1 (AtLRRAC1$^{48-205}$, AtLRRAC1$^{234-390}$, AtLRRAC1$^{426-583}$ and AtLRRAC1$^{1210-1365}$) were docked with ATP at their AC centres and a total of 18 solutions generated by AutoDock Vina (ver. 1.1.2) (Trott and Olson, 2010) for each fragment were then evaluated and expressed as percentage (see Figure 2B). Orientations and binding poses were analysed with the UCSF Chimera package (Pettersen et al., 2004). Chimera is developed by the Resource for Biocomputing, Visualization, and Informatics at the University of California, San Francisco (supported by NIGMS P41-GM103311). In docking simulations, all bonds in the ATP ligand were allowed to move but the protein was rigid. Docking orientations of ATP were evaluated based on previously determined "correct binding pose" e.g., in (Wong et al., 2015) and (Wong and Gehring, 2013). Specifically, the orientation of substrate ATP deemed favourable for catalysis was previously determined to be as follows: adenine head pointing towards position 1 of the motif and phosphate tail pointing towards position 14 of the motif. This was based on the rationale that: 1) position 14 of the AC motif is a highly conserved positively charged amino acid that binds phosphate strongly, 2) cation-binding amino acid is situated at 1-3 position downstream of the catalytic centre and is typically bound by divalent cations Mg^{2+}/Mn^{2+} in order to coordinate the phosphate of ATP, and 3) the catalytic roles of these highly conserved amino acids have been elucidated in ACs and GCs from bacterial and animal origins from which the AC motif was built (Gehring, 2010). Docking simulations consider both spatial and charge at the vicinity of the catalytic centre based on pre-determined grids that cover the catalytic centre entirely which we have set prior to docking experiments. Although different binding poses of ATPs with good binding affinities were obtained (e.g., between -2.6 to -6.6 kcal/mol), not all solutions show the "correct binding pose" deemed suitable for catalysis. However, if adequate number of simulations were performed, it will in theory generate statistically significant "correct binding pose" frequencies for proteins that are catalytically more active. Thus, we manually analysed the binding pose of each solution and take into consideration how frequent the software finds the “correct binding pose” instead of assessing the binding affinities. We found from 18 solutions, a significant preference for the "correct binding pose" for ACs with [DE] compared to those without. In AC2 and AC3 centres, even in instances where the

“correct binding pose” was obtained, part of the ATP substrate (adenine or phosphate) seems too far from the key amino acids at the AC centres as indicated by black arrows in the surface models. This approach was also adopted and have proved effective in previous structural evaluations of GC centres that investigated substrate preferences (Marondedze et al., 2015) and the effects of mutations to key residues at the catalytic centres (Al-Younis et al., 2018; Wheeler et al., 2017; Wong and Gehring, 2013).

2 Supplementary Table

Supplementary Table 1. Sequence-specific primers for amplification of the different AtLLRAC1 gene fragments harbouring the multiple AC catalytic centers.

AC Centre	Targeted Fragment	Forward Primer	Reverse Primer
AC1	$LRRAC1^{48\text{-}205}$	5′-GTTGCGGATGCTGATCAGAGAGCAGAACAT-3′	5′-GGTAGTCTTTCCAACCCCTGGCATACCAAC-3′
AC2	$LRRAC1^{234\text{-}390}$	5′-GCGGTTACAAAAGCAGTTCTGCAGGATATC-3′	5′-GAAATTCTTTGATACAGCATACCAGTCGTC-3′
AC3	$LRRAC1^{426\text{-}583}$	5′-GTCGATAGGGAGGAATTAGTACTCCTATGG-3′	5′-GTTTAACGATTTAGGCAAGTTGGTTATCTG-3′
AC4	$LRRAC1^{1210\text{-}1365}$	5′-GTCCCACAAGGAGGCCTTCCAACACCGAAA-3′	5′-GAGACAAGACAAAGGAGGAAGATCTTCATC-3′

In order to enhance efficiency of the cloning system, every nucleotide at the 5-end of each primer was deliberately made to be G (Invitrogen Corp., Carlsbad, USA).

Supplementary References

Al-Younis, I., Wong, A., Lemtiri-Chlieh, F., Schmöckel, S., Tester, M., Gehring C, et al. (2018). The *Arabidopsis thaliana* K^+-uptake permease 5 (AtKUP5) contains a functional cytosolic adenylate cyclase essential for K^+ transport. *Front. Plant Sci.* 9, 1645.

Gehring, C. (2010). Adenyl cyclases and cAMP in plant signaling - past and present. *Cell Commun. Signal.* 8, 15.

Marondedze, C., Wong, A., Thomas, L., Irving, H., and Gehring, C. (2017). *Cyclic nucleotide monophosphates in plants and plant signaling.* In: Seifert R, editor. Non-canonical Cyclic Nucleotides. Cham: Springer International Publishing, p. 87-103.

Wheeler, J. I., Wong, A., Marondedze, C., Groen, A. J., Kwezi, L., Freihat, L., et al. (2017). The brassinosteroid receptor BRI1 can generate cGMP enabling cGMP-dependent downstream signaling. *Plant J.* 91, 590-600.

Wong, A., Gehring, C., and Irving, H. R. (2015). Conserved functional motifs and homology modelling to predict hidden moonlighting functional sites. *Front. Bioeng. Biotechnol.* 3, 82.

Wong, A., and Gehring, C. (2013). The *Arabidopsis thaliana* proteome harbors undiscovered multi-domain molecules with functional guanylyl cyclase catalytic centers. *Cell Commun. Signal.* 11, 48.
